# Biochemical, physicochemical property and archaea community characteristics in casing soil of cultivating *Stropharia rugosoannulata*

**DOI:** 10.3389/fmicb.2025.1686476

**Published:** 2025-12-10

**Authors:** Jinjia Liu, Xiang Wang, Wenting Su, Jixiang Wang, Jinqiang Wu, Songhao Tian

**Affiliations:** 1 Department of Biochemistry, Basic Medical College, Changzhi Medical College, Changzhi, China; 2 Shanxi Biological Research Institute Co., Taiyuan, China; 3 Department of Physiology, Basic Medical College, Changzhi Medical College, Changzhi, China; 4 Department of Epidemiology and Health Statistics of Public Health and Preventive Medicine, Changzhi Medical College, Changzhi, China; 5 Department of Histology and Embryology, Basic Medical College, Changzhi Medical College, Changzhi, China; 6 Department of Medical Laboratory Science, Fenyang College of Shanxi Medical University, Fenyang, China

**Keywords:** *Stropharia rugosoannulata*, soil, enzyme activity, physicochemical properties, nutrient indicators, soil archaea

## Abstract

Mulching is very important in the cultivation of *Stropharia rugosoannulata* and helps in the formation of edible mushroom substrates. The physicochemical properties of soil are crucial for the growth process of edible mushroom substrates. In this study, we evaluated the enzyme activities, physicochemical properties, nutrient indicators, and archaeal community characteristics of soil during five stages of cultivation of *Stropharia rugosoannulata* (casing, mycelial, primordial, growing, and harvesting). The results showed that sucrase activity decreased significantly and then increased with the increase of cultivation time, and urease activity fluctuated and then decreased significantly. Catalase activity peaked at stage A5. Polyphenol oxidase activity was significantly higher at stage A2 and lowest at stage A4. Soil pH reached its lowest (7.130) at stage A5 after fluctuation. Soil total organic matter content was highest at stage A5 and reached maximum at stage A5 (8.357 g/kg). Organic nitrogen content was for was significantly higher in A4 stage at 0.276 g/kg. Organic content was significantly higher in A1 and A4 stages (0.401, 0.397 g/kg). The number and diversity of archaea in the soil gradually increased, and a total of 10 genera of archaea from six phyla were identified. The present study provides data on soil characteristics and archaeal communities in the overburden of *Stropharia rugosoannulata* at different stages of cultivation, which can contribute to the sustainable cultivation of *Stropharia rugosoannulata* and the reduction of live pollutants.

## 1 Introduction

*Stropharia rugosoannulata* (*S. rugosoannulata*), belongs to *Basidomycota, Hyme-nomycetes, Agaricales, Strophariaceae, Stropharia. S. rugosoannulata* are rich in mineral elements such as K, P, Zn, Mn, Cu, and Fe. It contains a variety of phenols, saponins, flavonoids, and other antioxidant substances, and the content of VC and taurine is as high as 53.1 mg/100 g and 81.5 mg/100 g (Lv et al., 2025).

Soil-covering cultivation is a commonly used method in the production of *S. rugosoannulata*. In the cultivation process, pre-treated soil is covered over the cultivation substrate to change the environmental conditions such as temperature, light, air, and moisture on the surface of the cultivation substrate, so as to make the *S. rugosoannulata* change from the nutritive growth to the reproductive growth stage. Soil physicochemical properties have a crucial influence on soil microorganisms and will change to some extent with the growth of the substrate. By measuring the activities of sucrase, urease, catalase, and polyphenol oxidase, we were able to assess soil carbohydrate degradation, nitrogen transformation efficiency, redox capacity, and organic matter oxidation potential, respectively. These enzymes may influence the transformation, uptake, and utilization of essential nitrogen elements in the soil, thereby affecting the growth of edible fungi during the casing cultivation process (Liu et al., 2024). The most prominent challenge of mushroom production from mulching is contamination by soil-derived mycorrhizal fungi. Incomplete soil sterilization introduces pathogenic microorganisms during the mulching process, which usually results in the infection of *S. rugosoannulata* with stray bacteria and consequent production losses.

Archaea are important microorganisms that play an important role in the cycling of carbon, nitrogen, and other substances in the soil (Dang et al., 2008). Archaea are tolerant and widely distributed in various environments, with a lower percentage and abundance in soil microorganisms than bacteria and fungi, and fewer related studies (Sugano et al., 2005). In agricultural systems, archaea are increasingly recognized as key players in nitrogen transformation processes, particularly through their role in ammonia oxidation—a critical step in the nitrogen cycle that directly influences nutrient availability for crop growth. Studies have shown that archaeal community abundance is positively correlated with soil organic matter and total nitrogen content, while archaeal diversity is positively associated with salinity and ammonium nitrogen levels (Zhao et al., 2022). In addition, the anaerobic methane-oxidizing bacterium *Candidatus Methylomirabilis sinica* in soil has been reported to independently mediate a complete denitrifying anaerobic methane oxidation process (Yao et al., 2024). Unlike bacteria and fungi, archaea exhibit remarkable resilience to environmental stresses, making them potential indicators of soil ecosystem stability under changing cultivation conditions. In the context of *S. rugosoannulata* cultivation, understanding archaeal community dynamics may provide insights into the microbial mechanisms underlying substrate conversion efficiency and pathogen suppression, which are crucial for sustainable mushroom production. The community structure and distribution of archaea are influenced by various physicochemical factors such as temperature, pH, and organic matter content (Gubry-Rangin et al., 2017). The interactions of microorganisms such as archaea may soil biochemical cycling processes are potentially important (Zhu et al., 2025). In recent years, there has been a steady increase in the market demand for *S. rugosoannulata*. The study of the physicochemical properties and the community structure of archaea in the mulch soil to determine the optimal ecological environment favorable for the growth of *S. rugosoannulata*, especially in the process of mulching cultivation, is of great theoretical and practical significance for elucidating the micro-ecological mechanism of the mulch soil of *S. rugosoannulata*, improving the yield of mushrooms as well as overcoming the limitations of the cultivation technology.

Cultivation of crops affects the composition of soil bacterial, archaeal, and fungal communities and alters the microbial community structure and species diversity (Wang et al., 2009). Some microorganisms in this process may have an impact on the cultivation of *S. rugosoannulata*. The structure and diversity of inter-root bacterial and fungal communities of different crops have been studied more by previous researchers, while the diversity and community structure of archaea have not been sufficiently studied. The findings of this study are expected to provide theoretical foundations for optimizing *S. rugosoannulata* cultivation practices through microbial management strategies. By elucidating the relationships between archaeal communities and soil functionality, we may identify key archaeal taxa that contribute to substrate efficiency and disease resistance, ultimately supporting the development of sustainable mushroom production systems with reduced reliance on chemical fertilizers and pesticides, and help growers to formulate a program for controlling the growth and development of *S. rugosoannulata*, and lay a foundation for promoting the large-scale production of *S. rugosoannulata*.

## 2 Materials and methods

### 2.1 Experimental design and soil sample collection

In May 2023, experiments were conducted on the cultivation of *S. rugosoannulata* in mulch in an edible mushroom greenhouse in Xiaodian District, Taiyuan City, Shanxi Province, China, with strains supplied by Shanxi Biological Research Institute Co. Loamy soil 2–3 cm thick was selected for mulching. The soil was watered every 3 days after mulching, and the water content was controlled at 60%−65%, the indoor temperature was maintained at 20 °C, the air humidity was 85%−90%, and the air was ventilated at noon every day during the cultivation process. Shade and cooling were strictly monitored during cultivation management.

Samples were collected at five cultivation stages, representing the soil-covering stage (A1), mycelial stage (A2), protoplasmic stage (A3), growth stage (A4), and mushroom emergence stage (A5) at days 0, 25, 40, 55, and 70, respectively. Samples were collected at a depth of 2 cm after removing the top soil layer. All overburden soil samples were consistently sampled using the five-point sampling method, where soil samples from five sampling points were randomly selected, and 100 g of soil was taken from each sampling point and mixed to form a mixed sample. Three replicates were set up for each growth stage and a total of 15 samples were obtained. After removing soil particles with a 2 mm sieve, the samples were quickly placed in sterile glass containers, brought back to the laboratory in an ice box, and stored at −80 °C.

### 2.2 Determination of soil enzyme activities

Soil enzyme activities were measured as indicators of microbial metabolic functions. The sucrase activity of the soil was determined by the colorimetric method of 3,5-dinitrosalicylic acid, urease by the colorimetric method of sodium phenol, catalase by the titrimetric method of potassium permanganate, all of which were carried out with reference to the method of Liu et al. (2024), and polyphenol oxidase by the colorimetric method of pyrogallic gallic acid, with reference to the method of Jiang and Penner (2021).

### 2.3 Determination of soil physicochemical properties and nutrient indicators

Soil physicochemical properties (pH, TOC) and nutrient indicators (AN, AP) were determined to assess the soil's basic chemical environment and fertility status. The pH of the soil samples was determined using a pH meter (soil-water mass ratio of 1:2.5), organic matter (TOC) was determined using potassium dichromate coupled with heat capacity method (Rukhovich et al., 2025), soil quick nitrogen (AN) content was determined using alkaline dissolution diffusion (ADD) method (Venkatesh et al., 2024), and the content of quick phosphorus (AP) was determined using an ultraviolet spectrophotometer (Hannet et al., 2021).

### 2.4 DNA extraction, amplification and sequencing of soil microorganisms

Genomic DNA of soil samples was extracted by CTAB. DNA concentration and purity were determined using TBS-380 and NanoDrop 2000, and DNA integrity was assessed by 1% agarose gel electrophoresis. DNA libraries were constructed using NEBNext^®^ UltraTM DNA Library Prep Kit, and macro-genomic raw data of archaea in the samples were sequenced using Illumina NovaSeq for macro-genomic sequencing. Removal of splice sequences and removal of low-quality (threshold ≤ 30) sequences from the raw data, removal of Reads with final read lengths less than 50 bp, comparison of Clean Data to the host genome using Bowtie2, and finally quality control by FastQC. The sequences were compared to the UniRef90 protein database using HUMAnN2 to obtain annotation information and relative abundance for each functional database (Suzek et al., 2007).

### 2.5 Statistical analysis

Soil enzyme activities, physicochemical properties, and microbial population data were analyzed by one-way analysis of variance (ANOVA) using IBM SPSS Statistics 20 software and tested for significant differences using the multiple comparison method (Duncan). Calculate the alpha diversity indices of the archaeal community using the “diversity” function from the vegan package in R.

Principal Coordinate Analysis (PCoA) based on Bray-Curtis distance was used to determine structural differences in bacterial and fungal communities in the overburden. Phyloseq with and ggplot2 R package (3.3.6) was used to perform principal coordinate analysis and plot PCoA.

Redundancy analysis (RDA) was conducted using CANOCO 5 to evaluate the effects of soil physicochemical properties on microbial communities. Correlation analysis between archaeal taxa and environmental factors was performed using the pheatmap package, and significant multivariate relationships were assessed with the envfit test, applying significance thresholds of *r*^2^ > 0.5 and *p* < 0.01. Species annotations and abundance information of all the samples at each taxonomic level, based on their abundance information within each sample was clustered based on their abundance information. Co-occurrence networks were analyzed using the Igraph program package in R in combination with Gephi software (Tan et al., 2022).

## 3 Results

### 3.1 Soil enzyme activities at different stages of cultivation of *S. rugosoannulata*

**Figure 1:**
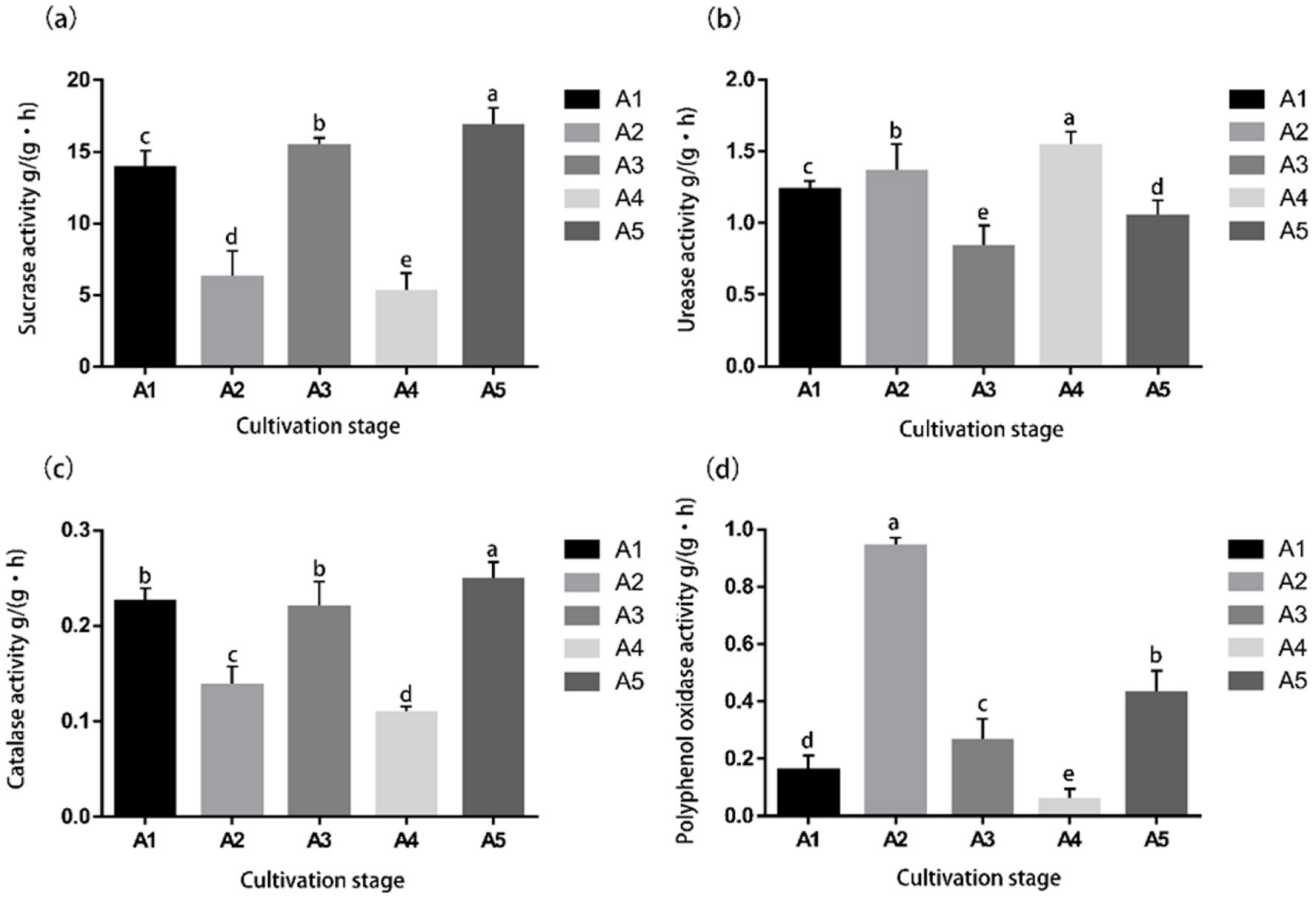
Changes of soil enzyme activity during the cultivation of *S. rugosoannulata*. **(a)** Sucrase activity of casing soil across five cultivation stages; **(b)** Urease activity of casing soil across five cultivation stages; **(c)** Catalase activity of casing soil across five cultivation stages; **(d)** Polyphenol oxidase activity of casing soil across five cultivation stages. Different letters meant significant difference among treatments at *p* < 0.05.

Soil enzyme activities varied significantly among the five mulch cultivation stages of *S. rugosoannulata* (Figure 1). During cultivation, sucrase activity was 13.880 g/(g-h) at stage A1 and declined significantly thereafter, showing fluctuations at different times and peaking at 16.72 g/(g-h) at stage A5 (Figure 1a). Soil urease activity increased significantly from 1.237 g/(g-h) at stage A1 and then decreased significantly to the lowest level of 0.837 g/(g-h) at stage A3 until it reached a peak of 1.533 g/(g-h) at stage A4 and then declined (Figure 1b). Catalase activity gradually fluctuated up and down from 0.226 g/(g-h) in the A1 period to 0.112 g/(g-h) in the A4 period, which was at its lowest, and then increased significantly to a peak of 0.253 g/(g-h) in the A5 period (Figure 1c). Polyphenol oxidase activity peaked at 0.895 g/(g-h) during the A2 period, then gradually decreased to the lowest level of 0.058 g/(g-h) during the A4 period, and rapidly increased to 0.433 g/(g-h) during the A5 period (Figure 1d).

### 3.2 Soil physicochemical indicators at different stages of cultivation of *S. rugosoannulata*

**Figure 2:**
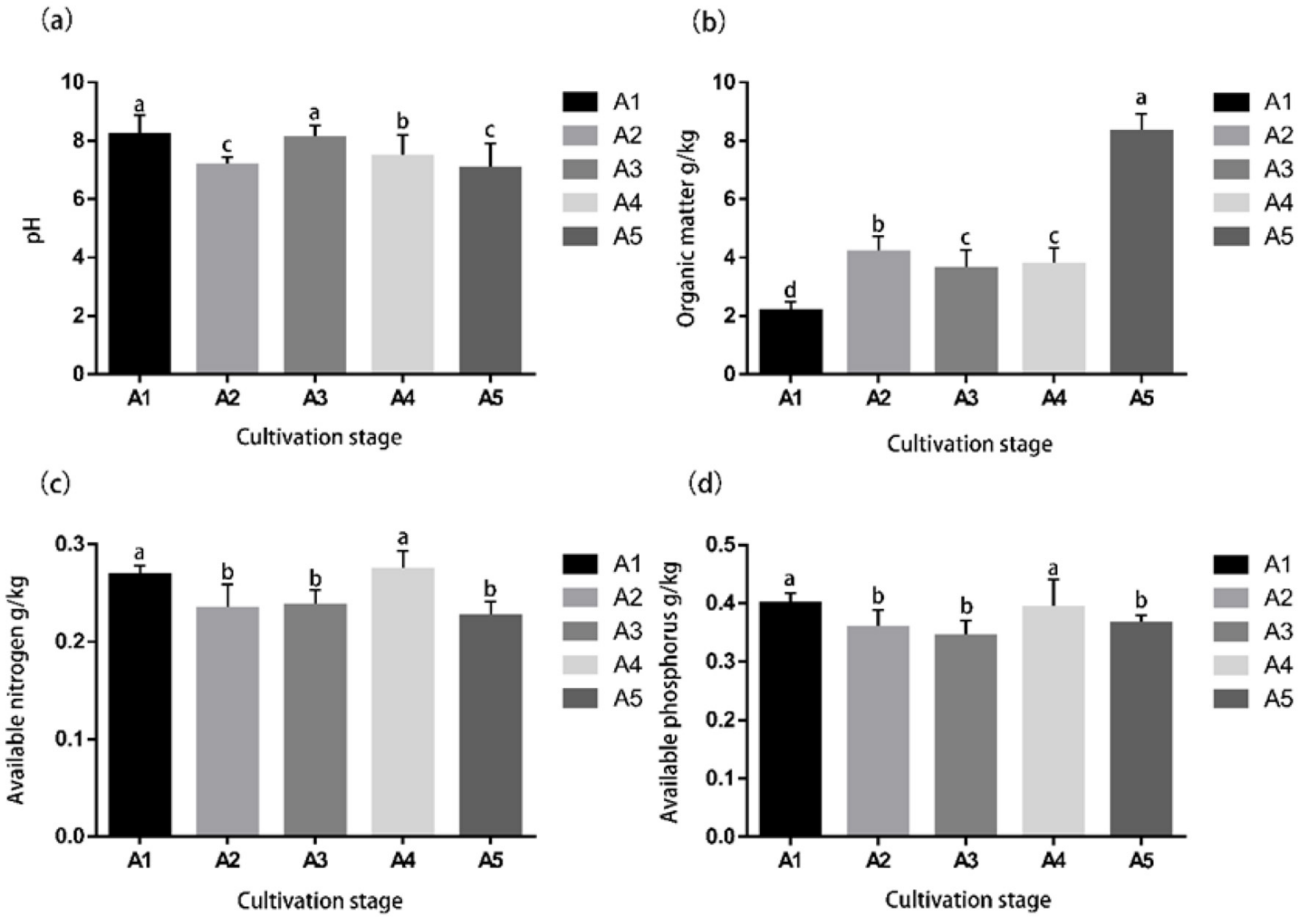
Physicochemical properties and nutrient indicators of the casing soil during the cultivation of *S. rugosoannulata*. **(a)** Casing soil pH across five cultivation stages; **(b)** Soil total organic matter content across five cultivation stages; **(c)** Available nitrogen (AN) content across five cultivation stages; **(d)** Available phosphorus (AP) content across five cultivation stages. Different letters meant significant difference among treatments at *p* < 0.05.

Various soil physicochemical properties and nutrient indicators were evaluated at different stages of mulching cultivation of *S. rugosoannulata* (Figure 2). Soil pH fluctuated back from a significant decrease of 8.21 at stage A1 to a minimum of 7.130 at stage A5. Soil organic matter TOC content increased significantly from 2.204 g/kg at stage A1 to 8.357 g/kg at stage A5. Organic nitrogen AN content was 0.271 g/kg at stage A1 and 0.276 g/kg at stage A4, which was significantly higher than that of other stages. Organic phosphorus AP content decreased significantly from 0.401 g/kg in stage A1 to 0.362 g/kg in stage A2 and 0.397 g/kg in stage A4, which was significantly higher than the other stages in A1 and A4 (A3: 0.347 g/kg; A5: 0.368 g/kg).

### 3.3 Effects of different stages of cultivation of *S. rugosoannulata* on the diversity of soil archaea

**Figure 3:**
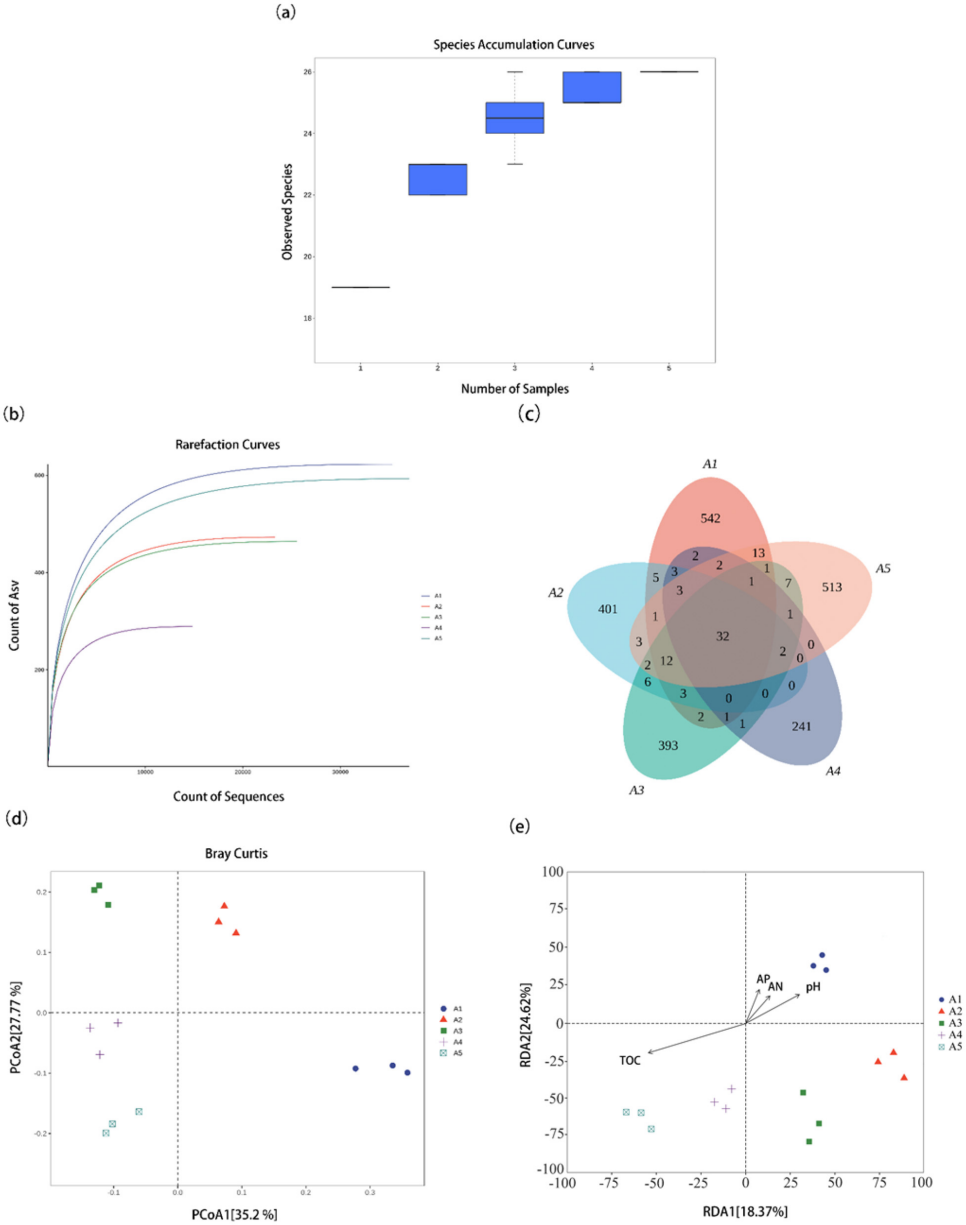
Effects of different stages of cultivation of *S. rugosoannulata* on the diversity of soil archaea. **(a)** Sample-based species accumulation curves (the horizontal coordinate is the sample size, and the vertical coordinate is the number of species observed by random sampling at this sample size); **(b)** Rarefaction curve for species of archaea (the horizontal axis represents the sequencing data volume, while the vertical axis shows the number of ASVs that can be counted based on this sequencing quantity); **(c)** OTUs of archaea communities in the soil at different cultivation stages; **(d)** Structure analysis of the archaea community in casing soil cultivation of *S. rugosoannulata* using Principal Component Analysis (PCoA); **(e)** Effects of the physicochemical properties casing soil on archaea communities (TOC, total organic carbon; AP, available phosphorus; AN, available nitrogen).

Species accumulation curves are used to characterize the trend of increasing species numbers with increasing sample size, which helps to study species composition and predict species richness in sample plots. Species accumulation curves for soil archaea (Figure 3a) increased sharply when sample sizes were small. As the sample size increases, the significant increase in the total number of archaeal species stops due to the addition of new samples. The curve gradually flattens and asymptotes, indicating that the sample size is sufficient for data analysis.

The Rarefaction Curve (RC) is used to assess whether the sequencing volume is sufficient to cover all microbial taxa in the sample and indirectly reflect the richness of species in the sample (Faith et al., 1987). At the species level, the minimum sample size was used for draw leveling and the dilution curve was plotted (Figure 3b). The results showed that the species range of the archaeal community was 284–616, and with the gradual increase in the number of samples of the archaeal community, the curve of the number of species tended to flatten out, which indicated that the depth of macro-genome sequencing in this study has basically covered all the species in the samples, and the data were This indicates that the depth of macro-genome sequencing in this study has basically covered all the species in the samples, and the data are representative and sufficient to reflect the diversity of the archaeal communities.

Based on the macro-genome sequencing results, the species diversity of soil archaea was counted. As can be seen from the figure, a total of 2193 OTUs of archaeal species were detected in the soils of five periods: A1, A2, A3, A4, and A5. The number of species detected in the five soils were 623, 473, 464, 289, and 593 OTUs, accounting for 28.4, 21.5, 21.1, 13.1, and 27.0% of the total number of archaeal species in the soil, respectively; the number of species OTUs among different forage land soil archaea among the species number OTUs decreased in the following order: A1 > A5 > A2 > A3 > A4 (Figure 3c).

The alpha-diversity indices of archaeal communities at stages A1–A5 and their statistical comparisons are presented in Table 1. The numerical variations and significance patterns clearly reflect the dynamic changes in community richness and evenness across cultivation stages. Species richness exhibited a significant increasing trend, rising from 4210 ± 28.14 at stage A1 to 5050 ± 38.65 at stage A5. Statistical analysis indicated significant differences between stages A1 and A2, A2 and A3, and A3 and A4, whereas no significant difference was detected between A4 and A5. This suggests that the rate of increase in species number gradually stabilized during the later stages of cultivation. The Chao1 index, an important estimator of species richness, showed a pattern highly consistent with the richness index, increasing continuously from 4520 ± 35.26 at A1 to 5350 ± 45.62 at A5. Significance testing revealed that all adjacent stages from A1 to A4 differed significantly, while A4 and A5 showed no significant difference. These results further confirm that potential species continued to accumulate throughout the cultivation process but tended to stabilize toward the later stages. Similarly, the ACE index increased from 4480 ± 32.18 at A1 to 5290 ± 40.73 at A5. Statistical analyses identified significant differences among stages A1, A2, A3, and A4, whereas no significant difference was observed between A4 and A5. This indicates that the archaeal community approached a dynamic equilibrium in richness and evenness during the later cultivation stages. Overall, these trends suggest that as cultivation progressed, improvements in habitat conditions and increasing structural completeness of the community provided a more suitable environment for species colonization and proliferation, ultimately contributing to the stabilization of community diversity.

**Table 1:** Alpha diversity analysis.

**Growth stage**	**Richness index**	**Chao1 index**	**Ace index**
A1	4210 ± 28.14^a^	4520 ± 35.26^a^	4480 ± 32.18^a^
A2	4350 ± 30.52^b^	4680 ± 38.75^b^	4620 ± 34.86^b^
A3	4620 ± 32.87^c^	4950 ± 40.12^c^	4890 ± 36.54^c^
A4	4810 ± 35.43^d^	5120 ± 42.37^d^	5060 ± 38.21^d^
A5	5050 ± 38.65^d^	5350 ± 45.62^d^	5290 ± 40.73^d^

Note: Values within the same column followed by different superscript letters (a–d) differ significantly at the 0.05 level (p < 0.05). Values sharing the same letter are not significantly different.

Based on the Bray–Curtis distance, PCoA was used to analyze the β-diversity of archaeal community structure in the overburden soil at different stages of the cultivation of *S. rugosoannulata*. The results showed that the *S. rugosoannulata* soil samples A4 and A5 were closer on the horizontal (35.2% of the explained variance) and vertical axes (27.77% of the explained variance) and dispersed from the other samples, suggesting that the archaeal community structure in the soil of *S. rugosoannulata* at stages A4 and A5 was similar. However, the archaeal communities of the other samples were dispersed on the horizontal and vertical axes of the PCoA, indicating significant differences in the archaeal community structure of the other overburden soil samples (Figure 3d). During the cultivation of *S. rugosoannulata*, the archaeal community structure in the casing soil exhibited a clear pattern of stage-specific succession. Samples from A4 and A5 clustered closely together in the PCoA plot, indicating a high degree of similarity in archaeal community composition during these periods. In contrast, samples from A1, A2, and A3 were widely dispersed along both the horizontal and vertical axes, suggesting that the archaeal community underwent rapid turnover during the early stages from casing to primordia initiation. Among these, A1 samples were farthest from the A4/A5 samples, revealing substantial differences in archaeal community structure between the initial casing stage and the fruiting stage. This transition from dispersed to clustered distributions reflects an ecological process in which the archaeal community shifts from rapid adaptation to a more stable state as *S. rugosoannulata* progresses from vegetative growth to reproductive development.

Redundancy analyses using pH, TOC, AN, and AP of overburden soils were performed to determine the effect of soil physicochemical properties on bacterial and archaeal communities during cultivation of *S. rugosoannulata*. Each point in the resultant graph represents a different sample, and the arrows represent different physicochemical properties; the length of the arrows represents the intensity of the effect of the physicochemical properties on the changes in the community; the angle between the arrows and the axes represents the correlation between the physicochemical properties and the axes; and the distance of the sample point to the arrows indicates the degree of the effect of the physicochemical properties on the samples. The results showed that organic matter had the greatest effect on the changes of archaeal communities. Toc had a significant and positive correlation on the archaeal communities of soil samples A4 and A5. pH, AP, and AN had a significant and positive correlation on the archaeal communities of soil sample A1 (Figure 3e). The total explanatory power of these factors on the differences in the archaeal community of soil at different stages of cultivation of *S. rugosoannulata* was 42.99%.

### 3.4 Community composition of soil microorganisms

**Figure 4:**
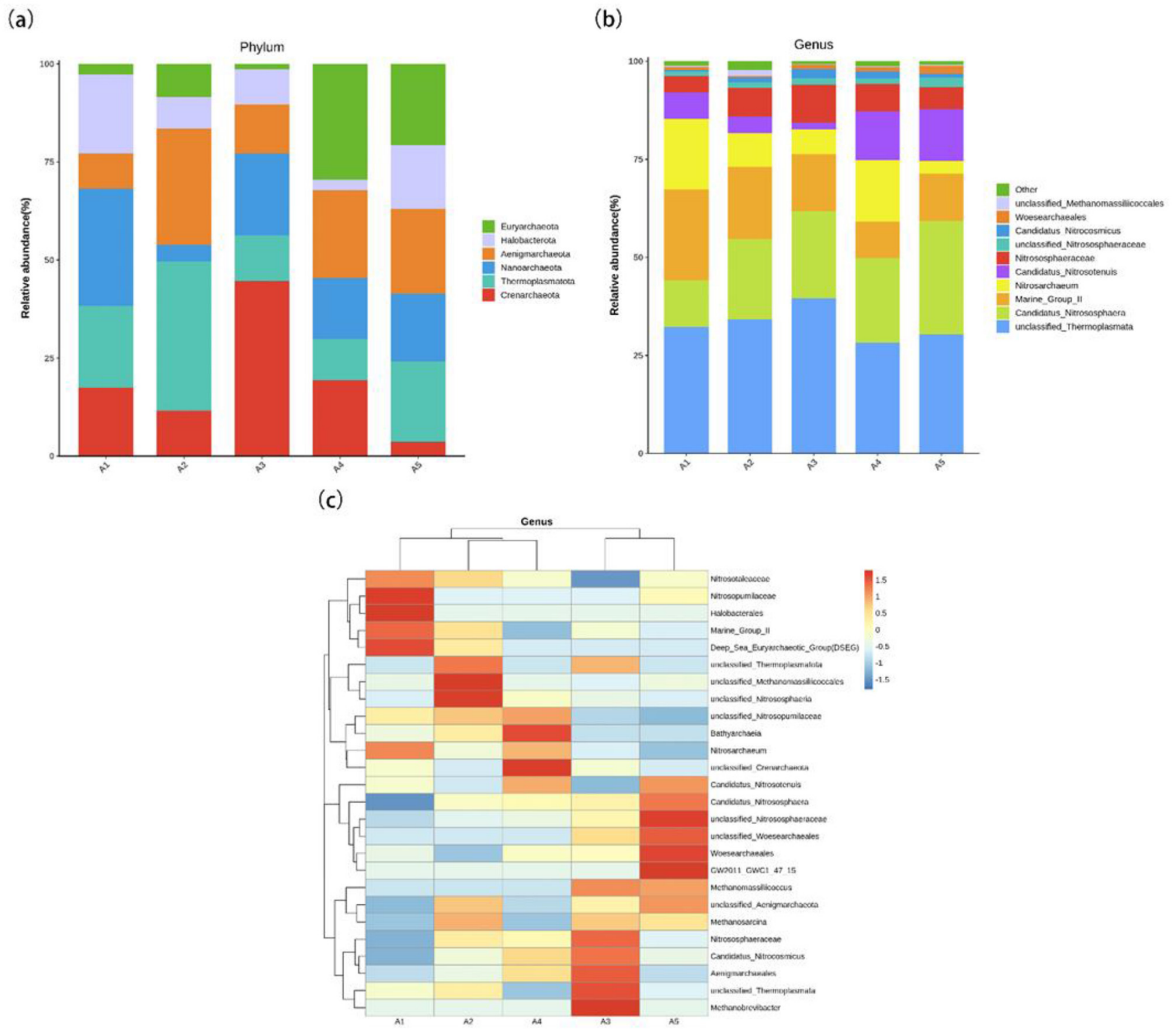
The composition of the archaeal community of soil microorganisms. **(a)** Archaea relative abundance at the taxa in the soil at different cultivation stages of *S. rugosoannulata*; **(b)** Archaea relative abundance at the genera in the soil at different cultivation stages of *S. rugosoannulata*; **(c)** Heatmap of soil archaea at the genus level.

High-throughput sequencing results showed that the top six archaeal phyla detected in the five soil samples were *Thermoplasmatota, Crenarchaeota, Aenigmarchaeota, Nanoarchaeota, Euryarchaeota*, and *Halobacterota*. *Thermoplasmatota* had the highest relative abundance, which first increased from 20.81% at A1 to a peak of 38.34% at the A2 period before decreasing and finally reaching 20.20% at A5, a pattern indicating that *Thermoplasmatota* was most active during the mycelial growth stage (A2). *Crenarchaeota* had the highest relative abundance at the A3 stage with 44.76%, which corresponds to the primordia formation stage, suggesting that its ammonia-oxidizing activity may provide the necessary nitrogen source for primordia differentiation. *Aenigmarchaeota* reached its highest relative abundance at A2 (29.12%), a period consistent with the rapid mycelial growth stage. The relative abundance of *Nanoarchaeota* peaked at A1 at 29.80%. *Halobacterota* reached its highest relative abundance at A1 at 20.20%, then decreased to 2.6% at A4 and rebounded to 16.31% at A5, indicating that *Halobacterota* was more active during the early casing and harvesting stages. The relative abundance of *Euryarchaeota* peaked at 29.6% in A4 (Figure 4a), reflecting the enhanced anaerobic conditions of the soil microenvironment during this stage.

At the genus level, 3 of the top 10 genera were unclassified. Unclassified_*Thermoplasmata* had the highest relative abundance of 28.30%−39.51%. The relative abundances of other genera were *Candidatus_Nitrososphaera*: 111.78%−28.86%, Marine_Group_II: 9.19%−23.24%, *Nitrosarchaeum*: 3.26%−17.89%, *Candidatus_Nitrosotenuis*: 1.64%−13.27%, Genus *Nitrososphaeraceae*: 4.07%−9.67%, unclassified_*Nitrososphaeraceae*: 1.16%−2.29%, *Candidatus_Nitrocosmicus*: 0.45%−2.41%, genus *Woesearchaeales*: 0.42%−1.94%, unclassified_*Methanomassiliicoccales*: 0.14%−1.54%, and the relative abundance of the remaining archaeal genera was 0.768%−2.22% (Figure 4b).

**Figure 5:**
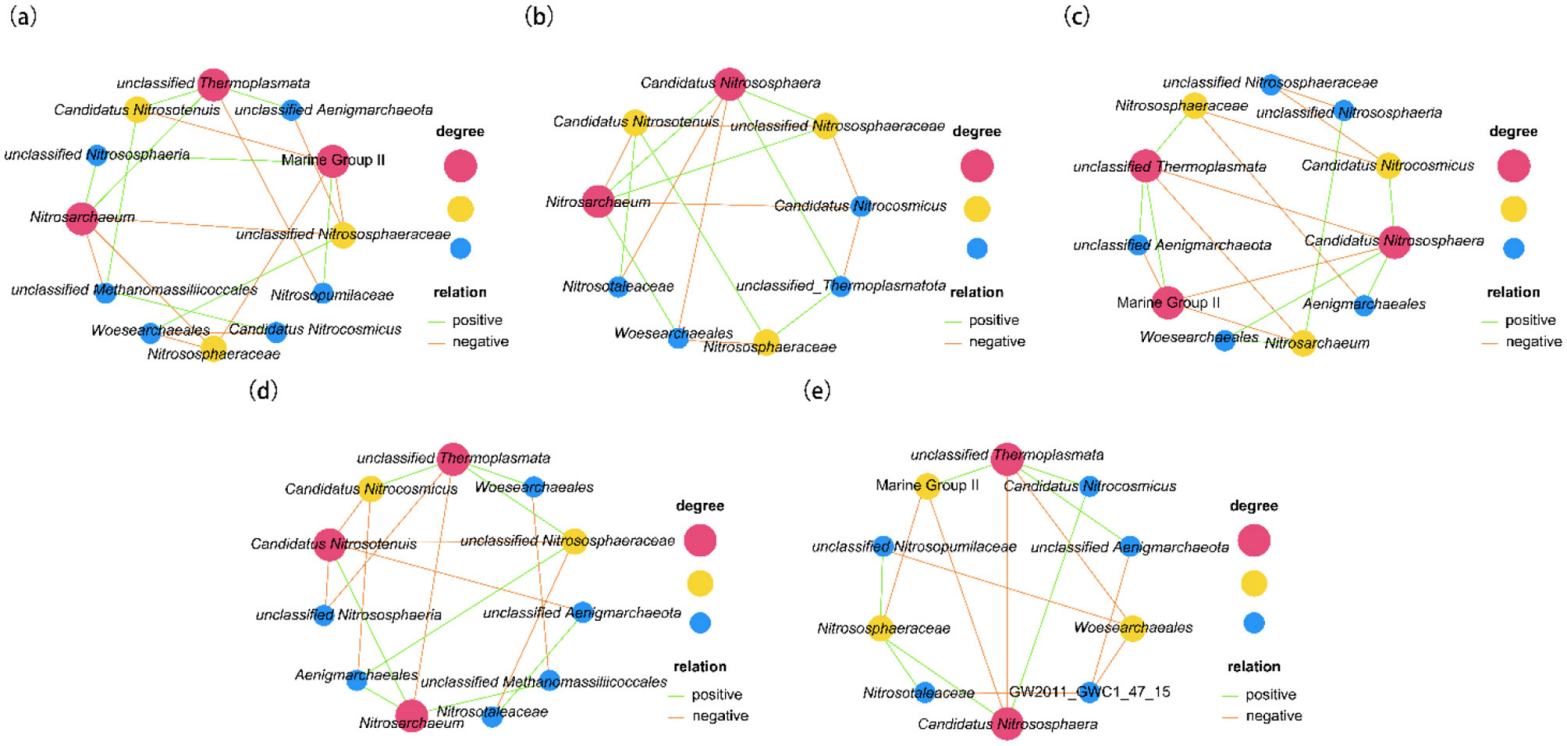
Co-occurrence networks of archaea genera at five cultivation stages of *S. rugosoannulata*. **(a)** casing, A1; **(b)** mycelial, A2; **(c)** primordial, A3; **(d)** growing, A4; **(e)** harvesting, A5.

The top 26 archaeal taxa were selected based on abundance and clustered at the genus level by their abundance in each sample. Heat maps were generated to visualize species concentrations for each sample (Figure 4c). For genus-level archaeal communities, the relative abundance of soil archaea varied across five phases. Marine_Group_II, *Nitrosarchaeum, Nitrosotaleaceae, Nitrosopumilaceae*, unclassified_Crenarchaeota, Deep_Sea_Euryarchaeotic_Group (DSEG), *Halobacterales* had the highest relative abundance in A1; unclassified_*Methanomassiliicoccales*, unclassified_*Nitrososphaeria*, unclassified_*Thermoplasmatota*, and *Methanosarcina* had the highest relative abundance in A2; unclassified_*Thermoplasmata, Nitrososphaeraceae, Candidatus_Nitrocosmicus, Aenigmar-chaeales, Methanomassiliicoccus*, and *Methanobrevibacter* had the highest relative abundance in A3; unclassified_*Nitrosopumilaceae*, and *Bathyarchaeia* had the highest relative abundance in A4; *Candidatus_Nitrososphaera, Candidatus*_*Nitrosotenuis*, unclassified_*Nitrososphaeraceae, Woesearchaeales*, unclassified_*Aenigmarchaeota*, GW2011_GWC1_47_15, and unclassified_*Woesearchaeales* had the highest relative abundance in A5. Heatmap analysis showed that key functional ammonia-oxidizing archaea (e.g., *Nitrosarchaeum, Candidatus Nitrososphaera*) exhibited higher relative abundances during the A1 and A5 stages, which may be associated with the increased demand for nitrogen transformation at the initial casing stage and the harvesting stage. In contrast, methanogenic archaea (e.g., *Methanosarcina, Methanobrevibacter*) displayed elevated abundances at the A3 stage, reflecting shifts in the soil microenvironment during the primordia formation phase. The sparc-based co-occurrence network showed that the interactions between some archaeal communities changed at different stages of *S. rugosoannulata* culture (Figure 5).

At stage A1, Marine Group II was significantly positively correlated with *Candidatus Nitrosotenuis, Nitrososphaeraceae*, and unclassified *Nitrosophaeraceae*; unclassified *Nitrosophaeraceae* was significantly positively correlated with unclassified *Aenigmarchaeota, Nitrosarchaeum* significantly positively correlated; *Nitrosopumilaceae* significantly positively correlated with unclassified *Thermoplasmata*; *Nitrososphaeraceae* significantly positively correlated with *Nitrosarchaeum, Woesearchaeales* significantly positively correlated; *Nitrosarchaeum* significantly positively correlated with unclassified *Methanomassiliicoccales*.

At stage A2, *Candidatus Nitrososphaera* was significantly positively correlated with *Nitrosotaleaceae* and *Woesearchaeales*; *Nitrosarchaeum* was significantly positively correlated with *Candidatus Nitrosotenuis, Candidatus Nitrocosmicus* was significantly positively correlated; *Candidatus Nitrosotenuis* was significantly positively correlated with unclassified *Nitrosophaeraceae*, unclassified *Thermoplasmatota*.

At stage A3, unclassified *Nitrososphaeria* was significantly positively correlated with *Nitrosarchaeum; Candidatus Nitrocosmicus* was significantly positively correlated with *Candidatuss Nitrososphaera; Candidatuss Nitrososphaera* is significantly positively correlated with *Aenigmarchaeales* and *Woesearchaeales*; unclassified *Thermoplasmata* is significantly positively correlated with *Nitrososphaeraceae* and unclassified *Aenigmarchaeota*, Marine Group II were significantly positively correlated; *Woesearchaeales* was significantly positively correlated with *Nitrosarchaeum*.

At stage A4, unclassified *Thermoplasmata* was significantly positively correlated with *Woesearchaeales*, unclassified *Nitrososphaeraceae*, and *Candidatus Nitrocosmicus*; unclassified *Nitrososphaeraceae* was significantly positively correlated with *Aenigmarchaeales*; unclassified *Aenigmarchaeota* was significantly positively correlated with *Nitrosotaleaceae*; *Nitrosarchaeum* was significantly positively correlated with unclassified *Methanomassiliicoccales, Aenigmarchaeales*, and *Candidatus Nitrosotenuis* significantly positively correlated.

At stage A5, unclassified *Thermoplasmata* was significantly positively correlated with Marine Group II, *Candidatus Nitrocosmicus*, and unclassified *Aenigmarchaeota*; *Candidatus Nitrososphaera* was significantly positively correlated with *Candidatus Nitrocosmicus, Nitrososphaeraceae* significantly positively correlated; *Nitrososphaeraceae* significantly positively correlated with *Nitrosotaleaceae*, unclassified *Nitrosopumilaceae*. The decrease in the number of nodes between the genera of Archaea indicates a decrease in correlation complexity.

## 4 Discussion

The use of different types of soil cover affects the rate of substrate formation, traits, yield, and bioconversion efficiency of edible mushrooms. In this study, soils used in five growth stages of cultivated *S. rugosoannulata* were selected as study materials. The community composition characteristics of soil archaea and their influencing factors during different cultivation periods were studied by measuring soil enzyme activities and physicochemical properties, and using high-throughput sequencing combined with various ecological and statistical analysis methods.

During the cultivation of *S. rugosoannulata*, the activity of sucrase, urease, catalase, and polyphenol oxidase may be affected by the activities of soil microorganisms, especially archaea. Soil plays a vital role in the ecosystem and is a sensitive indicator of soil biological traits, and during the cultivation of *S. rugosoannulata*, metabolites produced by the growth of the substrate altered the composition of soil nutrients and affected the diversity of the microbial community in the soil, or of some of the archaea therein. Soil pH significantly decreased and organic matter content significantly increased during the harvesting period, while both potassium nitride and fast-acting potassium content significantly decreased. This may be due to the fact that the dominant archaea in the soil absorbed and utilized ammonium nitrogen, nitrite, and other substances during the growth process causing corresponding changes in soil physicochemical properties (Zhu et al., 2025).

PCoA analysis showed that the A4 and A5 samples clustered together, whereas the samples from A1 to A3 were widely dispersed, indicating that the archaeal community structure became more stable during the fruiting stage, while rapid turnover occurred during the earlier stages. This pattern was highly consistent with the stage-specific changes observed in soil enzyme activities and physicochemical properties: sucrase activity peaked at A5, corresponding to the increased carbon demand during fruiting body maturation, while urease activity reached its maximum at A4, which is associated with intensified nitrogen metabolism during primordia differentiation.

Soil TOC, pH, AP, and AN has different effects on archaeal communities. Soil archaeal communities were most affected by TOC, which may be due to the fact that the organic matter in this soil is more specific for one or several archaea in the soil (Bale et al., 2019). In many soil samples, TOC was positively correlated with changes in archaeal communities, and the effect was more pronounced at later stages, indicating that the growth of archaea is highly dependent on organic matter.

In the present study up to 616 archaeal ASVs were identified. Among them, *Nitrosarchaeum* is a thermophilic, chemoautotrophic, and aerobic ammonia-oxidizing archaeon isolated and obtained from agricultural soils, which can tolerate high concentrations of ammonium nitrogen and nitrites (Jung et al., 2018); *Candidatus_Nitrosotenuis* is the only culturable, mesophilic member that derives energy by oxidizing ammonia and carbon and fixing carbon dioxide (Bale et al., 2019); *Woesearchaeota*, which accounts for a large portion of archaeal diversity, has previously been found in numerous natural environments (Tully et al., 2017; Liu et al., 2021) and has been found to be naturally enriched in lake water (Lake Dziani Dzaha; Liu et al., 2021), the *Woesearchaeota* archaeal communities in the degradation of starch, glycogen and sugar end products of lactic acid and acetic acid, they are the main intermediates in the interaction metabolism and symbiotic relationship (Cloarec et al., 2024). Unclassified_*Thermoplasmata* had the highest relative abundance of 28.30%−39.51%, and they may be involved in key nitrogen and carbon cycling functions affecting the growth of *S. rugosoannulata* (Cloarec et al., 2024). There were also significant differences in the community structure of soil archaea at different times. When the relative abundance of different archaea reached the maximum value at different stages of culture of *S. rugosoannulata*, it indicated that these microorganisms might play unique roles in the growth and development of *S. rugosoannulata* during these periods.

Soil physicochemical properties are key factors affecting microbial communities, showing dynamic changes, influencing microbial diversity and structure (Diamond et al., 2022), and affecting the production and quality of substrates. The RDA results showed that soil pH, organic matter, AN, and AP significantly affected the structure of bacterial communities. Soil pH and organic matter significantly affected the fungal community. Heatmap analysis revealed a close association between the spatiotemporal distribution of key functional taxa and mushroom growth and development. *Nitrosarchaeum* showed its highest abundance at the A1 stage, suggesting that it may initiate nitrification during the early casing period and provide accessible nitrogen for mycelial growth. *Candidatus Nitrosotenuis* was enriched at the A5 stage, and its ammonium tolerance (Bale et al., 2019) may help maintain nitrification activity under the high-nitrogen conditions characteristic of fruiting body maturation. *Woesearchaeales* exhibited abundance peaks at both A3 and A5, and this group has been reported to participate in glycolysis and syntrophic metabolism (Cloarec et al., 2024), potentially contributing energy through carbon metabolism during mushroom development. The successional patterns of these functional taxa were consistent with the increasing trend in soil TOC content, indicating that archaea may participate in carbon supply by degrading organic matter, thereby indirectly supporting mushroom biomass accumulation.

Further research is needed to understand how these physicochemical properties affect the quality of the *S. rugosoannulata* through microorganisms and to identify specific microbial species that may support the development of the protocorm and fruiting body, which will help to unravel the micro-ecological mechanisms of the *S. rugosoannulata* mulching soil and provide insights into large-scale production of *S. rugosoannulata*. This study provides new ideas for future work on microbial diversity and the culturability of Archaea.

## Data Availability

The original contributions presented in the study are publicly available. This data can be found here: [10.6084/m9.figshare.30827210].
